# The Association of Obesity and Overweight with Executive Functions in Community-Dwelling Older Women

**DOI:** 10.3390/ijerph20032440

**Published:** 2023-01-30

**Authors:** Marcelo de Maio Nascimento, Matthias Kliegel, Paloma Sthefane Teles Silva, Pâmala Morais Bagano Rios, Lara dos Santos Nascimento, Carolina Nascimento Silva, Andreas Ihle

**Affiliations:** 1 Department of Physical Education, Federal University of Vale do São Francisco, Petrolina 56304-917, Brazil; 2 Department of Psychology, University of Geneva, 1205 Geneva, Switzerland; 3 Center for the Interdisciplinary Study of Gerontology and Vulnerability, University of Geneva, 1205 Geneva, Switzerland; 4 Swiss National Centre of Competence in Research LIVES—Overcoming Vulnerability: Life Course Perspectives, 1015 Lausanne, Switzerland; 5 Multiprofessional Residence, Hospital das Clínicas of the Federal University of Minas Gerais, Belo Horizonte 30130-100, Brazil; 6 Department of Psychology, Federal University of Vale do São Francisco, Petrolina 56304-917, Brazil

**Keywords:** aging, vulnerability, body mass index, cognitive functions, obesity, overweight

## Abstract

Among the risk factors reported for cognitive decline, the literature highlights changes in body composition. Thus, the aim of the present study was to examine the relationship between obesity/overweight and executive functions in cognitively normal older adult women. This cross-sectional study included 224 individuals (60–80 years), stratified into normal weight (n = 45), overweight (n = 98), and obesity (n = 81). As outcomes, body mass index (BMI), waist circumference (WC), and Trail Making Test Parts A and B were assessed. We found positive correlations of BMI and WC with completion times of TMT-A and TMT-B, and a negative correlation of BMI and WC with education. ANCOVA showed an association between higher BMI and slower completion time of TMT-A, TMT-B, and ΔTMT (B-A). Impairment of executive functions of cognitively normal older women may be positively associated with obesity and negatively associated with years of education. The findings may contribute to designing strategies that make it possible to prevent cognitive decline in women during aging.

## 1. Introduction

In old age, it is important for individuals to have intact cognitive functioning in order to remain independent and to present adequate levels of health, quality of life, and wellbeing [1]. However, cognitive decline is strongly associated with advancing age [2,3]. When it comes to cognitive aging, several factors are associated with this process. Moreover, interindividual differences influence the set of alterations [4]. Thus, some people manage to maintain their cognitive functions at a conserved level for a relatively long time, and many are able to promote their cognitive abilities through specific interventions [5], while others experience a sharp decline [6].

Evidence has shown that there is an association between changes in body composition (i.e., obesity and overweight) and cognitive performance deficits in older adults [7,8,9]. In 2017, the Global Burden of Diseases found that since 1980, worldwide, the prevalence of obesity has doubled in more than 70 countries, as well as progressively increased in most other nations [10]. Due to this, obesity is considered an important public health problem worldwide [11], which in turn contributes to increased vulnerability, morbidity (e.g., cardiovascular diseases), and mortality. Furthermore, regardless of an individual’s body mass index (BMI) measurement, a large waist circumference (WC) poses a health risk [12]. A review study with a meta-analysis that included 72 studies (n = 4952 obese/overweight individuals; 8–77 years old) showed a high deficit in the performance of executive functions (EFs) in obese participants [13]. The authors also verified impairment of inhibition and working memory in overweight individuals. Moreover, there is evidence that highlights a longitudinal relationship between obesity and a subsequent decline in EFs [14]. On the other hand, evidence has shown that obesity-related cognitive impairment can be reduced in individuals who have adopted an active and healthy lifestyle.

Although previous studies have reported an association between obesity/overweight and cognitive impairment in the older adult population [6,15], there are still gaps in this area. A review study showed that there is little understanding of the risks caused by obesity/overweight to the cognitive performance of older adults, as well as little consensus about the protective factors during cognitive aging [7]. Moreover, the results presented by most investigations are heterogeneous, which requires the adoption of methodological approaches with adjustments for different covariates, especially in relation to years of education [8]. Another knowledge gap concerns the lack of information about the association between obesity and cognition in older adults living in developing countries, especially those in cities far from large urban centers [16]. Finally, it is estimated that in 2050, Brazil, where the present study was carried out, will have the fifth largest population in the world [17]. In this context, 19 million Brazilians will be octogenarians, a vulnerable group with high rates of multimorbidity, including obesity/overweight and cognitive impairment [6,18]. Moreover, among Brazilian older adults, the proportion of women is higher than that of men. This fact was considered as a multifaceted phenomenon, titled the feminization of aging [19]. Thus, the present study aimed to examine the relationship between obesity/overweight and executive functions in cognitively normal older adult women.

## 2. Methodology

### 2.1. Study Design

This is a cross-sectional study. Members of the present study resided in the city of Petrolina, located in the Northeast region of Brazil. The study was approved by the Research Ethics Committee of the Federal University of Vale do São Francisco (Number 2866.845). The evaluations were carried out by specially trained field staff, between 2018 and 2019, in the Laboratory of Education, Culture and Movement (UNIVASF).

### 2.2. Sample Size Calculation

An a priori calculation for sample size using G*Power3 (Heinrich Heine University Düsseldorf, Düsseldorf, Germany) was performed considering the following: analysis of covariance (ANCOVA), f = 0.30, alpha error probability = 0.05, power = 0.87, number of groups = 3. A minimum of 220 individuals were required to achieve adequate statistical power. Finally, 224 subjects were included in the analysis.

### 2.3. Participants and Eligibility

Members of this study were recruited by telephone from the register of the University of the Third Age (U3A), which is a project for the older adult population offered by the Federal University of Vale do São Francisco [20]. Participants were on the waiting list for a vacancy to participate in educational and/or sports activities offered by this U3A. Considering that most of the older adults in this U3A were female, our study exclusively includes women. Initially, 242 individuals were invited to participate in the study. Of these, 224 (92.6%) accepted the invitation and completed all assessments. Inclusion criteria were as follows: age ≥ 60 years; not participating at the time of the assessments or having participated in the last six months of any regular cognitive or physical–cognitive training program; and achieving the following scores in the Brazilian version of the Mini-Mental State Exam: 20 points for illiterates, 25 points for individuals with education between 1 and 4 years, 26.5 points for those aged 5 to 8 years, 28 points for 9 to 11 years of education, and 29 points for those with more than 11 years of education [21]. As exclusion criteria, we adopted having severe neurological diseases (such as Parkinson’s, Alzheimer’s, dementia) or not completing all assessments. All participants were informed about the objectives and risks of the study. Finally, those who agreed to participate signed a consent form as per the Declaration of Helsinki. Participants in this investigation did not receive any financial compensation.

### 2.4. Measurements

#### 2.4.1. Main Characteristics of the Participants

Through a questionnaire, a set of information was collected, including age, sex (1 = men, 2 = women), years of education, comorbidities, marital status ((1) single, (2) married/common-law, (3) divorced, (4) separated, (5) widowed), and the number of types of medication consumed per day. We also included questions about monthly family income. The responses were classified by the number of monthly minimum wages (SM) earned by the family in 2019 (1 MW = BRL 1100.00). Possible answers were as follows: (1) < MW, (2) 1–2 MW, (3) 3–4 MW, (6) ≥ 5 MW. To present the Brazilian MW results, we use US dollars, converting the values according to the average quotation of the US dollar in 2019.

#### 2.4.2. Assessment of Executive Functions

The Trail Making Test (TMT) was used to assess the performance of divided attention, visuomotor tracking, and cognitive flexibility. The TMT is a psychomotor test originally developed as part of the US Army Individual Testing Battery [22]. In TMT Part A, a processing speed visual scanning task was performed [23]. Thus, the participant was asked to draw lines, as quickly as possible, and sequentially connect a set of consecutively numbered circles (from 1 to 25), posted randomly on a page. While in Part B, the participant was subjected to a measure of cognitive flexibility [24]. The task consisted of connecting the same number of circles; however, the displayed sequence required alternating numbers and letters (1, A, 2, B, 3, C, etc.). Both exams were timed and we analyzed the time in seconds. Our analysis also considered the score obtained by the difference between the times of Part A being subtracted from Part B (i.e., B-A), defined as ΔTMT. The score determined by the ΔTMT served to control the performance of the TMT B for the effect of processing speed and motor speed. This result is considered an accurate measure for EF, superior to the single performance of Part B [24]: higher difference scores indicate poorer ability to switch tasks.

#### 2.4.3. Anthropometric Measurements

Weight and height were collected using an anthropometric scale and a Welmy stadiometer calibrated for 0.1 cm and 0.1 kg [25], with a maximum capacity of 150 kg. During the assessment, participants were instructed to wear light clothing and remain barefoot. BMI was used as a measure of body weight (adjusted for height). BMI was defined as (weight (kilograms))/(height (m)^2^). The BMI classification was based on World Health Organization (WHO) criteria [12]: underweight (BMI < 18.5 kg/m^2^), normal weight (18.5–24.9 kg/m^2^), overweight (25–29.9 kg/m^2^), and obesity (≥30.0 kg/m^2^). WC was measured with the aid of a flexible measuring tape. The measurement (diameter) was taken 2 cm below the navel, with the participants standing. During the exam, the tape remained in contact with the skin, and the subcutaneous tissue was not compressed. The WC selection criterion used the cutoff point of ≥88 cm [26]. All measurements were performed by a single specialist employee in the health area, previously trained and with long experience in administering the exams.

#### 2.4.4. Covariates

Taking into account that the number of years of formal education completed by individuals is positively correlated with the level of their cognitive functions throughout adulthood [27,28], we consider years of education as a potential covariate for the analysis. Moreover, age was considered as a further covariate.

### 2.5. Statistical Analysis

Data were analyzed using SPSS for Windows version 22.0. Data normality was verified by the Shapiro–Wilk test. Participants were stratified into three BMI groups (normal weight, overweight, obesity) and into two WC groups (normal, overweight). Thus, BMI and WC were considered as independent variables, while EF was analyzed as a dependent variable. Comparisons between groups regarding continuous variables (demographic characteristics, clinical measures, education, medications, and MMSE) were performed using unilateral analyses of variance (ANOVAs). Pairwise comparisons were performed using the Mann–Whitney U test, with Bonferroni corrections. Between-group comparisons for categorical variables (hypertension, diabetes mellitus) were calculated using the chi-square test. Pearson’s correlation coefficients (*r*) were used to test the relationships between the dependent variables (TMT-A and TMT-B) and the independent variables (age, BMI, WC, and education in years). The interpretation of Pearson’s correlation coefficients (*r*) was as follows: 0.10 = small, 0.30 = medium, and ≥0.50 = large [29]. Intergroup comparisons of BMI and WC with time to complete TMT-A, TMT-B, and ΔTMT/B-A were performed using an analysis of covariance (ANCOVA). The findings were presented in two models: Model 1 contains unadjusted data; in Model 2, the data were controlled for the two covariates (years of education and age). In the present study, education was classified into 4 levels: 0 = illiterate, 1 = 1–4 years of education, 2 = 5–8 years of education, 3 = more than 8 years of education. In cases where an overall group difference was significant, post hoc Bonferroni tests were used to determine the precise locus of any significant differences observed. The significance level adopted in all cases was α = 0.050.

## 3. Results

A total of 224 older women were included in the analysis; the mean age was 65.69 ± 3.70 years. Regarding BMI, none of the individuals were classified as underweight. The prevalence of normal weight, obesity, and overweight was 20.0%, 43.8%, and 36.1%, respectively. Table 1 shows the main characteristics of the groups. Significant differences (*p* > 0.050) were found for all anthropometric measures (weight, height, BMI, and WC). Based on the assessment of the MMSE instrument, members of the three groups indicated preserved cognitive performance of 26.28 ± 1.86 points (*p* = 0.066). The average time of formal education of the study participants was 2.32 ± 3.51 years (*p* < 0.001). Regarding marital status, the three groups showed a prevalence of married or common-law (*p* ≥ 0.050). When asked about household income (*p* ≥ 0.050), the vast majority reported values between 2 and 3 minimum wages (USD 211.00–420.00). The number of types of medication consumed daily was 3.51 ± 1.77 (*p* = 0.002). Among the self-reported comorbidities, the most prevalent was hypertension (33.4%); however, only diabetes mellitus showed a statistical difference (*p* < 0.001).

Regarding the strength and direction of the association between the variables (see Table 2 for an overview), a positive, large, and significant correlation was found between BMI and WC (*p* = 0.004), and a positive, medium, and significant correlation was found between BMI and TMT-A (*p* = 0.008), TMT-B (*p* = 0.006), and TMT B-A (*p* = 0.004). Positive, medium, and significant associations were identified between WC and TMT-A (*p* = 0.002), TMT-B (*p* = 0.007), and TMT B-A (*p* = 0.005). Years of education showed a negative and large association with BMI (*p* = 0.004), and a negative, medium, and significant association with the following variables: WC (*p* = 0.008), TMT-A (*p* = 0.009), TMT-B (*p* = 0.003), and TMT B-A (*p* = 0.007). Age showed negative, small, and significant associations with WC (*p* = 0.022); negative, medium, and significant associations with BMI (*p* = 0.012); and negative, medium, and significant associations with the following variables: education (*p* = 0.007), TMT-A (*p* = 0.032), TMT-B (*p* = 0.028), and TMT B-A (*p* = 0.004). Finally, a positive, large, and significant association was indicated between TMT-A and TMT-B (*p* < 0.001), TMT-A and TMT B-A (*p* < 0.001), and TMT-B with TMT B-A (*p* < 0.001).

Table 3 shows the unadjusted analysis of covariance (Model 1) revealing a slowness trend in the performance of EFs for a high BMI: TMT-A (F(2,221) = 85.27, *p* = 0.041), TMT-B (F(2,221) = 49.31, *p* = 0.130), and ΔTMT (B-A) (F(2,221) = 52.93, *p* = 0.670). Bonferroni’s post hoc tests indicated no difference in the TMT-B and ΔTMT (B-A) examination for those classified as overweight when compared to others classified as normal weight and obese (*p* > 0.050).

Analyses adjusted for education and age (Model 2) revealed that there was a significant association of BMI with the performance of EFs, i.e., slowness during the execution of the cognitive test: TMT-A (F(2,220) = 58.01, *p* = 0.028), TMT-B (F(2,220) = 28.44, *p* = 0.027), ΔTMT (B-A) (F(2,220) = 48.13, *p* = 0.048). Following the order of the results of the previous model, Bonferroni’s post hoc tests indicated no difference between the analyzed variables.

Regarding the measurement of WC (Table 4), the unadjusted analysis (Model 1) showed that with the increase in obesity (WC) there was a slower completion time in the TMT-A (F(1,221) = 15.68, *p* = 0.012) and TMT-B (F(1,221) = 4.30, *p* = 0.039), but not in ΔTMT (B-A) (F(1,221) = 0.71, *p* = 0.400). Attesting to the slowness in performing the cognitive assessment, the adjusted analyses (Model 2) indicated a negative and significant relationship between abdominal obesity (CC) and TMT-A performance (F(1,221) = 12.38, *p* = 0.002), but it was not significant for the TMT-B (F(1,221) = 2.30, *p* = 0.130) and ΔTMT (B-A) (F(1,221) = 0.182, *p* = 0.670).

## 4. Discussion

The present study investigated the relationship between obesity and executive function performance in a group of cognitively normal older women. Our findings indicated that the TMT-A and TMT-B instruments are complementary measures, indicating a positive, medium, and significant association with BMI and WC. These findings are in line with previous research suggesting a negative link between increased body fat and executive function performance in old age [7,8,13]. Unadjusted analysis showed that participants with poor EF had significantly higher BMI than those with good EF performance. These results are consistent with neuroimaging tests used to investigate the relationship between obesity and the maintenance of cognitive functions in old age [30,31]. The results indicated a significant relationship of overweight and obesity with a longer completion time for TMT-B, but not for TMT-A and ΔTMT. Non-significant intergroup results may be indicative of the absence of deficits in EF performance [24]. After controlling for confounders (education/years and age), the time to perform the TMT-B test was attenuated; however, the same did not occur for the TMT-A and ΔTMT (B-A). The findings partly evidenced the protective role of education (compensatory mechanism) against cognitive impairments [32]. Our results showed, more specifically, the notable influence that years of education play in protecting against cognitive impairments in association with adiposity. Moreover, the finding considers increasing age as a relevant factor for cognitive impairments [33,34].

Although BMI is not sovereignly related to executive dysfunction, its significant interrelation with age suggests that obesity-related EF deficits may intensify with the aging process even in a cognitively normal population [35,36]. Neuroimaging tests have indicated that obesity plays a role in the development of connectivity deficits between different brain regions [13], hence in task-related prefrontal cortical dysfunction. These neurostructural changes included a reduction in cortical volumes and a consequent decrease in several subdivisions of the frontal cortex, such as the superior, middle, and inferior frontal gyrus and orbitofrontal cortex [37,38]. On the other hand, neuroimaging tests also suggested that short-term obesity (developed in old age) would not be as detrimental to cognition as obesity obtained in middle age [39]. This shows the need for lifelong follow-up studies to qualify the understanding of the influence of obesity on structural and functional brain aging.

In the present study, unlike in Part A, used to assess sustained attention (concentration), participants with overweight or obesity needed more time to perform the task in TMT-B, responsible for examining alternating attention [40]. The results of the adjusted analysis corroborated the outcome of an investigation carried out with Brazilians (n = 318; 18–81 years), which pointed to years of education and age as being among the factors responsible for the worsening of performance in the TMT-A and TMT-B exams [41]. It is worth noting that the relationship between obesity/overweight and cognition is contradictory [9]. The fact comes from the relative imprecision that the BMI offers to measure adiposity: with aging, the individual’s body composition changes [42]. Thus, there are doubts about whether obesity is a cause or a consequence of cognitive dysfunction, knowing that there may also be a bidirectional relationship in this process [43]. From this perspective, evidence suggested that weight gain is partially associated with a neurological predisposition marked by a reduction in EFs.

WC is not only considered an effective measure of central adiposity [44,45], but it has also been linked to cognitive impairment [46]. Our raw analysis fully corroborated this idea. Thus, participants with greater abdominal adiposity showed longer visual search time and motor speed (TMT-A), as well as lower cognitive flexibility and task-switching skills (TMT-B). On the other hand, controlling for years of education and age indicated significant differences only in Part A of the TMT test. The protective role of education against cognitive impairments [32] is a possible explanation for this result or suggests that perhaps in the population evaluated, WC is not related to performance on the TMT-B.

Over the years, studies have shown that the damage to cognitive functions caused by adiposity would not be exclusively mediated by clinical factors, such as type 2 diabetes, hypoglycemic episodes, and hyperlipidemia [43,47]. One theory is that the relationship between obesity and EF deficit comes from low-grade inflammation in obesity [48,49]. Systemic and central neuroinflammatory processes are accompanied by an elevation of circulating pro-inflammatory cytokines (i.e., IL-10 and TGF-β) [50]. This type of central inflammation has been observed in individuals who received high-fat diets, as well as in a diet-induced obesity model [51]. The literature highlights that the neuroinflammatory response linked to obesity occurs in different structures of the central nervous system, such as the cerebellum, amygdala, cerebral cortex, and hypothalamus [52,53]. In practice, inflammation can migrate from peripheral tissue to brain regions such as the cortex and/or hippocampus, affecting fundamental cognitive processes such as learning and memory [54].

Deterioration of brain functionality through obesity has also been suggested to be due to another low-grade systemic inflammation mechanism, namely insulin resistance [55]. Insulin is an important hormone for neurophysiological processes involved in cognitive functions [56]. However, obesity can cause chronic hyperinsulinemia and/or resistance to peripheral insulin, inducing hypoinsulinemia in the brain, with consequent reduction of the glutamate receptor [57]. In turn, insulin resistance potentiates hippocampus atrophy, impairing the neurophysiological processes underlying learning and memory [58]. On the other hand, obesity can be reversed by the adoption of healthy lifestyle habits (e.g., diet, regular physical exercise), reducing insulin resistance, and preventing inflammatory processes and vascular dysfunctions, included among the possible mechanisms responsible for the relationship between obesity and cognitive impairment [55]. Previous studies have also suggested that persistent low weight may be a risk factor for decreased EF performance [59,60]. This can result from health problems or a dysregulation in the secretion of the hormone corresponding to anorexia, causing cognitive disorders.

Another explanation for the association between obesity and a deficit of EFs has been proposed by the immune model of failed self-regulation [61]. In this, it is assumed that differences in EFs may predispose individuals to overweight. The argument is that human behavior is determined by an interaction between the impulsive system and the executive control system [62,63]. Thus, individuals with low executive control would be more susceptible to eating fatty foods and, consequently, to weight gain. On the other hand, individuals with effective cognitive control would show protection against developing obesity [64].

Our study has some limitations: First, due to the cross-sectional design, our findings do not allow us to make inferences about the causality of the relationship between obesity/overweight and the performance of EFs among healthy older women throughout life. Second, we consider as a potential limitation of our investigation the non-inclusion of brain images, as well as the use of only one test to examine the performance of EFs. Third, we only included two measures (BMI and WC) as parameters for obesity, and these measures may be insufficient indicators of cardiometabolic health. It is known that in advanced age, obesity must be adjusted to body composition [65]; therefore, it is suggested that future studies include complementary measures. This is important because body composition is made up of body mass (skeletal muscle mass), fat mass, and fat-free mass. All of these factors affect the central nervous system, which is responsible for controlling cognition, including EFs. Fourth, although our findings were controlled by years of study and indicate that low education, more specifically cognitive impairment, was strongly associated with obesity, it is noteworthy that among individuals classified as normal BMI and overweight, we found participants with 5 or more years of study, while among the obese, all were illiterate or had 1 to 4 years of study. Fifth, the sociodemographic characteristics of the three groups were different from each other, which suggests a particularity of this population to be investigated. Sixth, we cannot exclude that other residual confounding factors may be involved in the relationship between overweight/obesity and impaired EFs; examples include (1) eating habits, (2) socio-environmental issues specific to the region where the study participants live, (3) sedentary lifestyle/physical activity level, and (4) race. Seventh, we also cannot rule out poor sleep quality or sarcopenia; both are common among older adults and may have influenced the results. Moreover, sleep disturbances and muscle wasting have been associated with changes in brain function, including an increased risk of Alzheimer’s disease [66,67].

## 5. Conclusions

The impairment of EFs was positively associated with obesity and negatively associated with years of education. Comparatively, the BMI measure was superior to the WC measure for identifying higher-order cognitive deficits in cognitively normal obese older adult women. The mechanisms underlying the findings of the present study need further investigation. Although more research is needed to understand the underlying mechanisms and bidirectional relationships between adiposity and cognition, our results may help in the design of different strategies to prevent cognitive decline in aging as well as the development of new approaches capable of improving neurocognitive skills, including EFs, attention, and memory. Moreover, by increasing levels of cognitive functions, it is possible to influence the lifestyle of older adults, preventing weight gain. Due to the lack of information in this important field of health and aging, especially on the cognitively normal older Brazilian population, we suggest that further studies be carried out, including longitudinal follow-ups.

## Figures and Tables

**Table 1:** Main characteristics of the sample.

Variable	Normal Weight (n = 45)	Overweight (n = 98)	Obesity (n = 81)	*p*-Value
Age (years)	65.69 ± 3.70	66.26 ± 4.27	65.37 ± 4.03	0.344
Weight	55.58 ± 4.66	67.74 ± 4.70 ^a^	80.64 ± 7.48 ^a,b^	<0.001
Height	157.13 ± 5.46	157.61 ± 4.96	155.11 ± 4.10 ^a,b^	0.028
BMI (kg/m^2^)	22.53 ± 1.70	27.26 ± 1.37 ^a^	33.51 ± 2.89 ^a,b^	<0.001
WC (cm)	82.63 ± 3.96	94.20 ± 2.10 ^a^	102.74 ± 9.78 ^a,b^	<0.001
MMSE	26.28 ± 1.86	26.28 ± 1.88	26.27 ± 1.85	0.066
Education level				<0.001
Illiterate	1 (2.2)	1 (1.0)	20 (24.7) ^a,b^	
1–4 years	21 (46.7)	51 (52.0) ^a^	61 (75.3) ^a,b^	
5–8 years	5 (11.1)	39 (39.8) ^a^	-------	
Over 8 years	18 (40.0)	7 (7.1)	-------	
Marital status n (%)				
Married/common-law	44 (97.7)	96 (98.0)	80 (98.8)	0.528
Widowed	1 (2.3)	2 (2.0)	1 (1.2)	
Household income, n (%)				
USD ≤ 210.00	4 (8.9)	5 (5.1)	4 (5.0)	0.621
USD 211.00–420.00	40 (88.9)	91 (92.9)	75 (92.6)	
USD 421.00–630.00	1 (2.2)	1 (1.0)	1 (1.2)	
USD 631.00–840.00	-----	1 (1.0)	1 (1.2)	
USD ≥ 841.00	-----	-----	-----	
Medication				0.002
1–4 types n (%)	33 (73.3)	67 (68.4) ^a^	39 (48.1) ^a,b^	
>4 types n (%)	12 (26.7)	31 (31.6) ^a^	42 (51.9) ^a,b^	
Comorbidities				
Diabetes mellitus				<0.001 ^†^
Yes n (%)	7 (15.6)	54 (55.1)	23 (28.4)	
Hypertension				0.169
Yes n (%)	24 (53.3)	48 (49.0)	51 (63.0)	

Data are expressed as mean (M) ± standard deviation (SD) or frequency (%); kg = kilograms; cm = centimeters; MMSE = Mini-Mental State Examination; BMI = body mass index; kg/m^2^ = kilograms/square meter; WC = waist circumference; *p* < 0.050 = ANOVA; ^a,b^ *p* < 0.050 = Bonferroni’s corrections; **^†^** *p* < 0.050 = chi-square test. The formation of the three groups was based on BMI cut-off points.

**Table 2:** Pearson correlations of scores on time to complete TMT-A and TMT-B with age, BMI, WC, and education.

Variable	BMI	Age	WC	Education	TMT-A	TMT-B
Age	−0.300 *					
WC	0.560 **	−0.180 *				
Education	−0.500 *	−0.370 **	−0.350 *			
TMT-A	0.450 **	−0.320 *	0.360 **	−0.380 **		
TMT-B	0.450 **	−0.400 *	0.310 **	−0.370 **	0.680 ***	
TMT B-A	0.480 **	−0.432 *	0.380 **	−0.412 **	0.701 ***	0.711 ***

BMI = body mass index; WC = waist circumference; TMT-A = Trail Making Test Part A; TMT-B = Trail Making Test Part B; * *p* ≤ 0.050; ** *p* < 0.010; *** *p* < 0.001.

**Table 3:** Comparisons of executive function performance in different BMI groups.

Variable	Normal Weight (n = 45)	Overweight (n = 98)	Obesity (n = 81)	*p*-Value
Model 1				
TMT-A	58.78 ± 12.37	64.85 ± 7.43	77.56 ± 11.02	0.041
TMT-B	152.95 ± 29.54	159.31 ± 48.57	213.10 ± 42.55	0.130
ΔTMT (B-A)	94.17 ± 57.43	94.46 ± 26.32	135.55 ± 42.53	0.670
Model 2				
TMT-A	59.57 ± 11.68	65.24 ± 7.71	78.68 ± 10.95	0.028
TMT-B	156.66 ± 28.60	160.65 ± 48.38	216.27 ± 37.55	0.027
ΔTMT (B-A)	97.10 ± 55.46	95.41 ± 24.55	137.60 ± 36.67	0.048

TMT-A = Trail Making Test Part A; TMT-B = Trail Making Test Part B; ΔTMT (B-A) = Delta Trail Making Test; Model 1 = analyses unadjusted; Model 2 = analyses adjusted for education (years) and age.

**Table 4:** Comparisons of executive function performance in waist circumference groups.

Variable	Normal (n = 89)	Abdominal Obesity (n = 135)	*p*-Value
Model 1			
TMT-A	63.50 ± 1.33	68.33 ± 1.08	<0.012
TMT-B	171.60 ± 5.19	179.48 ± 4.21	0.039
ΔTMT (B-A)	108.10 ± 4.74	111.15 ± 3.85	0.400
Model 2			
TMT-A	64.19 ± 1.25	69.88 ± 1.01	0.002
TMT-B	174.20 ± 4.87	183.76 ± 3.95	0.130
ΔTMT (B-A)	110.01 ± 4.63	108.88 ± 5.78	0.670

Note: TMT-A = Trail Making Test Part A; TMT-B = Trail Making Test Part B; ΔTMT (B-A) = Delta Trail Making Test; Model 1 = analyses unadjusted; Model 2 = analyses adjusted for education (years) and age.

## Data Availability

The data presented in this study are available upon request from the corresponding author.
